# Application of healthcare failure mode and effect analysis in controlling surgical instrument packaging defects

**DOI:** 10.1038/s41598-022-24282-7

**Published:** 2022-11-16

**Authors:** Liangying Yi, Yanhua Chen, Ruixue Hu, Juan Hu, Wei Pan

**Affiliations:** 1 Department of Sterile Processing Nursing, West China Second University Hospital, Sichuan University/West China School of Nursing, Sichuan University, Chengdu, Sichuan China; 2 Key Laboratory of Birth Defects and Related Diseases of Women and Children (Sichuan University), Ministry of Education, Chengdu, Sichuan China

**Keywords:** Health care, Risk factors

## Abstract

Surgical instrument packaging defects may affect the safety of medical care and patients and waste the hospital workforce, material resources, and financial resources. This study explored the application of healthcare failure mode and effect analysis in controlling surgical instrument packaging defects. We retrospectively evaluated the packaging process of 183,642 surgical instruments packaged in our hospital during January–June 2020 using the healthcare failure mode and effect analysis. Besides, we used a decision tree model to determine the steps requiring improvement and formulate the improvement measures. We applied the improvement measures to 190,231 surgical instrument packs packaged in our hospital during July–December 2020. Based on the healthcare failure mode and effect analysis, we compared the packaging defect rates before and after adopting the improvement measures. Of the 183,642 packs selected before adopting the improvement measures, 98 defects occurred, with a defect rate of 0.053%. However, of the 190,231 packs selected after adopting the improvement measures, 22 defects occurred, with a defect rate of 0.012%. The defect rate of surgical instrument packaging handled by the central sterile supply department staff was significantly reduced (χ^2^ = 50.822, *P* = 0.001) after adopting the improvement measures. Using the medical failure mode and effect analysis method to control the defects in surgical instrument packaging can effectively reduce the packaging defect rate, ensuring patient safety.

## Introduction

The central sterile supply department is the heart of the hospital’s cleaning, disinfection, and sterilization, including the logistics center for the supply and turnover of sterile items. The quality of work directly affects the safety of medical care and patients, whereas surgical instrument packaging defects waste the hospital workforce, material resources, and financial resources^1^. Based on reports, the packaging defect rate accounts for 47.95% of the work quality defects in the central sterile supply department^2^. Therefore, the central sterile supply department faces an urgent problem in reducing the instrument packaging defects. Healthcare Failure Mode and Effect Analysis (HFMEA) is a risk assessment and evaluation method recommended by the United States Joint Committee on Evaluation of Health Care Organizations in the medical field. It is a forward-looking and predictive risk management method that can be used to supervise high-risk nursing processes, identify and correct risk factors, and prevent mistakes in the first place^3–5^. At present, there are various ways to reduce the instrument packaging defect rate, although most of them can be rectified. However, methods for proactive risk management analysis and propose corrective measures are still rare. We applied the HFMEA to control the instrument packaging defects and achieve better results.

## Methods

### Study setting

A total of 373,873 surgical instrument packs packaged in the central sterile supply department of our hospital during January–December 2020 were selected as the study objects. Inclusion criteria: only the commonly used surgical instrument packs, such as cesarean section, delivery, laparoscopic, and hysteroscopy instrument packs, were selected. Exclusion criteria: the oversized packs (larger than 30 cm × 30 cm × 50 cm) or the overweight packs (more than 7 kg) were omitted from this study.

Of the 373,873 surgical instrument packs, 183,642 packaged during January–June 2020 were classified into the control group, and 190,231 packaged during July–December 2020 were classified into the experimental group. The quality controllers evaluated the instrument packaging quality and input the information of the packs to the surgical instrument tracking system.

### Data collection

We collected data about the number of the packs in the control and experimental groups and the packs with packaging defects in the two groups from the surgical instrument tracking system to compare the instrument packaging defect rates before and after adopting the improvement measures based on the HFMEA.

### Packaging quality criteria

Packaging was considered adequate when all of the following requirements were met: (1) the categories, number, and specification of the instruments in the pack were correct. For example, a cesarean section surgical instrument pack contains 1 double-ended ragnell retractor (30 cm), 8 pairs of curved head toothed ring forceps (26 cm), 1 thyroid retractor (20 cm), 1 pair of toothed forceps (18 cm), 6 pairs of curved hemostatic forceps (18 cm), etc. (2) the instruments met the functional demand. All instruments in the pack were in normal function. The articulation joints were flexible, and the instruments did not become bent. (3) the packaging achieved the desired quality in cleanliness. All instrument surfaces, articulation joints, and grooves were visibly clean, and there were no blood stains, dirt, or limescale in the instruments. (4) appropriate packaging materials were used. The Tyvek® medical packaging materials, produced by DuPont de Nemours, Inc., were used for low-temperature plasma sterilization; the medical non-woven fabric was used for autoclave sterilization. In addition, the size of the packaging materials must match that of the instruments. (5) the instruments were packaged using the proper packaging method: the pack containing multiple instruments was double wrapped using two layers of wrap; the paper-plastic pouches or paper pouches were used for single instruments. (6) the external chemical indicators, such as external sterilization indicator tapes, were placed on the outside of every pack to monitor sterilization. (7) sealing was adequate. Complete packaging information was input into the surgical instrument tracking system.

### Packaging defects

The packaging defects meant that the packaging quality did not meet the criteria. The defects included an incorrect number of instruments, wrong instrument specification, functional defects, undesired quality in cleaning, inappropriate packaging materials, improper packaging methods, external chemical indicator missing, and defective sealing.

### The implementation process of healthcare failure mode and effect analysis

#### Choose high-risk processes and team formation

Packaging is a complex process involving multiple steps, and packaging defects are the most critical issue. Nevertheless, packaging defects are high-risk processes via the analysis of department quality control data and team interviews. Thus, the HFMEA team was established: the head nurse of the department served as the supervisor; the head nurse served as the team leader; the rest of the staff completed the teamwork according to the division of labor.

#### Sorting out the related processes of packaging quality defects

**Figure 1:**
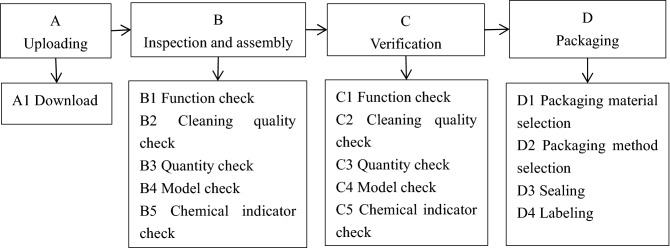
Exploded diagram of the packaging process.

The team members finally determined the primary approach of instrument packaging through discussion: unloading, inspection and assembly, verification, and packaging. Simultaneously, the sub-processes were also determined, as shown in Fig. 1.

#### Hazard analysis and decision tree analysis of device packaging process

List potential failure modes: The possible failure modes of the instrument packaging process were made based on the “brainstorming method” (Table 1).

**Table 1:** Screening of high-risk links in failure modes of packaging defects.

Process steps	Possible failure mode	Severity	Failure incidence	Risk priority number	If the decision (take action or stop) index was greater than 8, the action would be stopped, and the reason should be indicated
**A Unloading**					
A1 Download	A1a Unloading error	1	4	4	Stop
	A1b Drop	1	2	2	Stop
	A1c Omission	1	1	1	Stop
**B Inspection**					
B1 Function check	B1a Not checked	4	4	16	Continue
	B1b Missed	2	·2	4	Stop
	B1c Misjudgment	1	1	2	Stop
	B1d Wrong inspection method	2	3	6	Stop
B2 Cleaning quality check	B2a Not checked	1	4	4	Stop
	B2b Missed	1	4	4	Stop
	B2c Misjudgment	1	1	1	Stop
	B2d Did not see clearly	2	3	6	Stop
	B2e Wrong inspection method	2	2	4	Stop
B3 Quantity check	B3a More	4	4	16	Stop (verification and packaging would be eliminated in the future)
	B3b Less	4	4	16	Stop (verification and packaging would be eliminated in the future)
B4 Model check	B4a Model missing	2	2	4	Stop
	B4b Species missing	4	1	4	Stop
	B4c Incorrect model identification	1	1	1	Stop
B5 Chemical indicator check	B5a Not put in	4	4	16	Stop (verification and packaging would be eliminated in the future)
	B5b Put too much	4	1	4	Stop
	B5c Indicator selection error	2	1	2	Stop
**D Packaging**					
D1 Packaging material check	D1a Type selection error	1	1	1	Stop
	D1b Specification or model error	1	2	2	Stop
	D1c Packaging material quality defects	2	2	4	Stop
D2 Packaging method check	D2a Wrong	2	1	2	Stop
D3 Sealing	D3a Loose packaging	3	4	12	Continue
	D3b Indicating tape did not meet the criteria	1	1	1	Stop
D4 Labeling	D4a The label was printed incorrectly	4	4	16	Stop (verification and packaging would be eliminated in the future)
	D4b Label pasting error	4	1	4	Stop
	D4c Incorrect new label	2	1	2	Stop

Hazard Index score/risk priority number (RPN): We performed a hazard index score/RPN for all listed failure modes. According to the theory of failure modes, severity (S) and failure probability (P) form a hazard index. The total score is 1–16 points. When the score is ≥ 8 points, it is considered that this link will cause significant harm to the process and determined as a high-risk failure link^6,7^ (Table 1).

**Figure 2:**
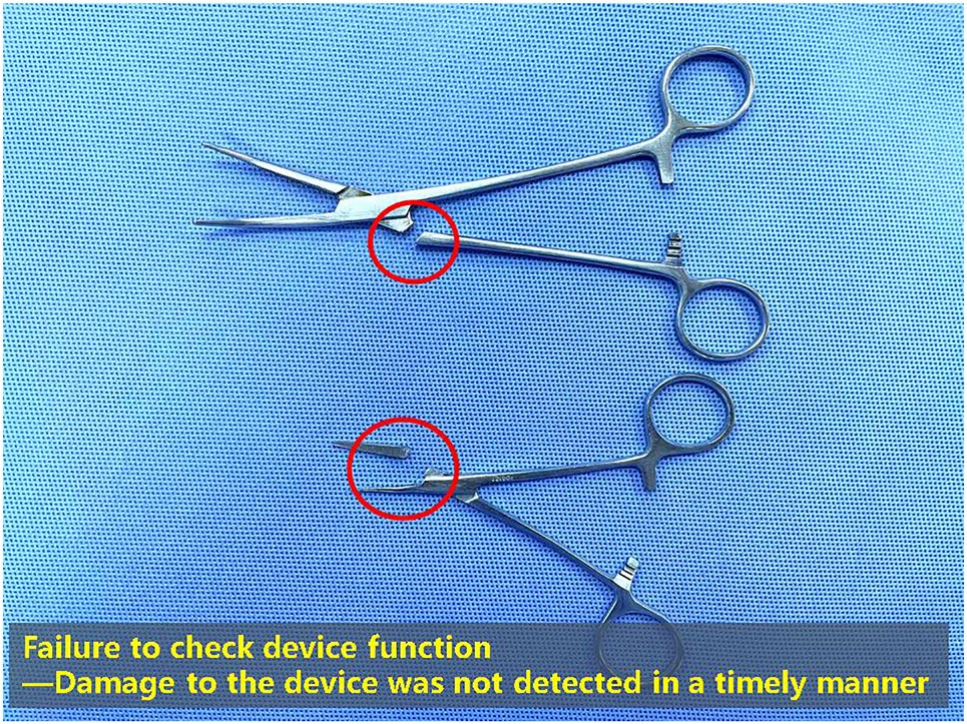
Failure to check device function.

**Figure 3:**
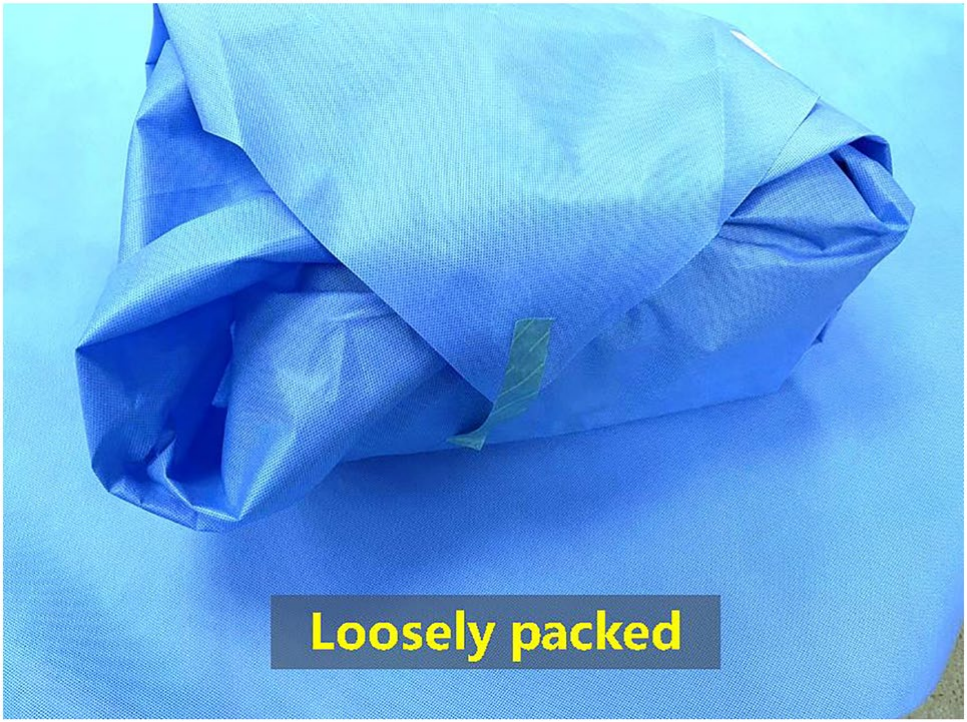
Loose device packaging.

Use decision tree analysis to determine whether to formulate improvement measures: The identified high-risk failure links were further incorporated into the judgment decision tree analysis: “whether it is the only weakness”, “whether effective control measures are formulated”, and “whether the danger can be detected”. Finally, it was decided to take improvement measures on the device with undetected damages (Fig. 2) and loose device packaging (Fig. 3)^8^ (Table 1).

#### Plan actions and measurements

Using the characteristic factor diagram (fishbone diagram) to analyze the reasons for the failure to check the instrument function and the loose instrument packaging, we found the root cause and formulated corresponding action strategies.

Improvement measures for the instruments with undetected damages during packaging: (1) We altered the mode of nurse training to meet the needs of different nurse levels. The problem-based learning (PBL) model was used, and the training was done through situational exercises so that the nurses could master the relevant knowledge of the instrument function. Furthermore, assessments were performed before and after the training to evaluate the effectiveness of the training. The plan-do-check-act (PDCA) cycle was used to continuously track the training effect and improve nurses’ familiarity with professional knowledge about instrument functions^9^. (2) A standard operating procedure (SOP) was produced for functional inspections of various instruments, and the reference drawings of the instruments were compiled. In turn, the inspection of the instrument function became standardized and intuitive, and staff compliance was improved. Simultaneously, all departmental staff must understand the importance of the functional status of commonly used surgical instruments to the operation and understand how to check the functions of various instruments^10^. (3) The frequency of down collection was increased from four to six times. Simultaneously, the number of mobile personnel with flexible shifts according to the amount of surgery was increased to prevent undetected damage caused by a large number of instruments collected in a short period. (4) The inspection system for packaging positions and standardized inspection methods were improved. Furthermore, the verification process was straightforward, including verification based on the exchange list before packaging, verification based on label details during packaging, strict double-person verification, and verification of label information outside the package and sealability after packaging.

Improvement measures for loose device packaging: (1) Central sterile supply department industry standard did not specify the length of the indicator adhesive tape for sealing, which was highly random. For this reason, according to scientific proof, our department designated catheterization kits, vaginal inspection kits, suture kits, etc. as small packs. The length of the adhesive tape was 10 cm for 1 piece of pack. The abortion packs, cervical examination packs, and the general laparoscopic instrument packs were considered medium-sized packs. The length of the sealing tape was 12 cm, and 3 sealing tapes were used for every medium-sized pack. Fetal removal instrument packs, uterine instrument packs, bath towel packs, etc. were considered large packs. The length of the sealing tape was 15 cm, and 4 sealing tapes were used for every large pack. For the oversized or overweight pack, one more corresponding tape should be added based on the actual situation. (2) The SOP for special-shaped item packaging should be provided for packaging handler to follow when they assembled the special-shaped items; the packaging process was improved further by reducing the occurrence of loose device packaging.

### Statistical methods

SPSS 17. 0 statistical software was used for analysis, and the count data were analyzed using χ^2^ test. *P* < 0.05 indicated a statistically significant difference.

## Results

Table 2 presents a comparison of the instrument packaging defect rate before and after adopting the improvement measures based on the HFMEA.

**Table 2:** Comparison of the defect rates in surgical instrument packaging.

Groups	Number of the surgical instrument packs	Number of packs in conformity with packaging criteria	Number of the packs with packaging defects	Defect rate (%)
Control group	183,642	183,544	98	0.053
Experimental group	190,231	190,209	22	0.012

χ^2^ = 50.822; *P* = 0.001.

After adopting the improvement measures based on the HFMEA, the instrument packaging defect was significantly reduced. A statistically significant difference was found, as shown in Table 2.

## Discussion

### Risk assessment and analysis of instrument packaging

Figure 1 depicts the main process of instrument packaging after the team members jointly confirmed it: unloading, inspection and assembly, verification, and packaging. Simultaneously, the main process was divided into sub-processes. Besides, the team members listed all possible failure modes for each sub-process and calculated the hazard index based on the severity of failure mode and frequency with which it could occur. For the hazard index ≥ 8 points, more actions were required. According to Table 1, the hazard index of four circumstances was higher than 8; namely, instrument damage undetected, an insufficient number of instruments, an excess number of instruments, and loose instrument packaging. Thus, these four circumstances required further analysis.

### Analysis of decision-making actions

The HFMEA decision tree indicates three issues: if the failure mode and step will cause significant damage to the system; if this item is the only weakness or key in the entire process; if there is no effective measure to control the occurrence of the failure mode, the appropriate actions must be taken. In both the instrument verification and packaging, an insufficient number of instruments and an excess number of instruments had corresponding inspection systems that could effectively prevent incidents. However, there was no effective way to control this failure mode if the device function was not checked and packaging was loose. Therefore, the actual cause of instrument packaging process failure was that the device function was not being checked, or the device packaging was loose; thus, appropriate measures were taken to address the problem.

### Analysis of the decline in packaging defect rate

The results presented in Table 2 show that after the implementation of medical failure mode and effect analysis management, the instrument packaging defect rate has dropped from 0.053 to 0.012%. This research first sorts out the instrument packaging process and decomposes the primary process and sub-processes to clarify the entire process. Subsequently, the hazard analysis was performed in the healthcare failure mode using the hazard score matrix and effect analysis. The primary reasons for the packaging defects were also found. The team developed various intervention plans based on critical factors, improved the instrument packaging process, and more standardized the behavior of instrument packaging personnel. The analysis of healthcare failure modes and their effects were focused on prevention. Based on the early prediction of relevant risk factors, targeted and comprehensive nursing measures were developed to improve deficiencies in previous operations and improve the level of operation, thereby preventing the occurrence of operational risk events^11–14^. During the analysis, it was discovered that when the instrument was packaged, the function of the instrument was not tested, and the packaging was loose, posing significant hidden risks to the entire instrument packaging process. Therefore, it is necessary to strengthen the management of related links to reduce packaging defects. If the instrument packaging handler makes an error in packaging, it is usually attributed to a personal mistake or behavioral error. However, it is not difficult to find that most errors are caused by behavioral deviations caused by imperfect processes or systems, accounting for about 70% of the total^15,16^. Therefore, after using the healthcare failure mode and effect analysis, the manager modified his or her previous practice in attributing errors to the person packaged the instruments. Instead, it examines all aspects of the entire device packaging process by producing various device functional inspection SOP, particular device packaging SOP, packaging standards for various sterile packages, and a complete inspection system. More attention should be paid to selection of packaging materials and containers. The sizes of the packaging materials and containers should match that of the instruments. Finally, optimizing the device packaging process reduces packaging quality defects and effectively improves the packaging quality of medical devices to ensure surgical safety for patients^17^.

### Limitation

This study is a single-center study, with a short data collection time and short observation time after the experiment. We hope that the data used here can be expanded, and the observation time can be extended further to evaluate the effect of the investigation in the future.

## Conclusions

In the current work of the central sterile supply department, packaging quality defects are a key issue in post work. This study has shown that tools such as failure mode and effect analysis can perform a detailed analysis of the complex packaging process, outline potential failure modes, and emphasize which risks are most worrying to guide improvement efforts and focus on high-risk tasks.

## Data Availability

The datasets used and/or analyzed during the current study are available from the corresponding author on reasonable request.

## Abbreviations

HFMEA: Health care failure mode and effect analysis
RPN: Risk priority number
SOP: Standard operating procedure
